# Superior radiation-resistant nanoengineered austenitic 304L stainless steel for applications in extreme radiation environments

**DOI:** 10.1038/srep07801

**Published:** 2015-01-15

**Authors:** C. Sun, S. Zheng, C. C. Wei, Y. Wu, L. Shao, Y. Yang, K. T. Hartwig, S. A. Maloy, S. J. Zinkle, T. R. Allen, H. Wang, X. Zhang

**Affiliations:** 1 Department of Materials Science and Engineering, Texas A&M University, College Station, TX 77843; 2 Materials Science and Technology Division, Los Alamos National Laboratory, Los Alamos, NM 87545; 3 Department of Nuclear Engineering, Texas A&M University, College Station, TX 77843; 4 Department of Materials Science and Engineering, Nuclear Engineering Program, University of Florida, Gainesville, FL 32611; 5 Department of Nuclear Engineering, The University of Tennessee, Knoxville, TN, 37996, USA; 6 Department of Engineering Physics, University of Wisconsin, Madison, WI 53706, USA; 7 Department of Electrical and Computer Engineering, Texas A&M University, College Station, TX 77843; 8 Department of Mechanical Engineering, Texas A&M University, College Station, TX 77843

## Abstract

Nuclear energy provides more than 10% of electrical power internationally, and the increasing engagement of nuclear energy is essential to meet the rapid worldwide increase in energy demand. A paramount challenge in the development of advanced nuclear reactors is the discovery of advanced structural materials that can endure extreme environments, such as severe neutron irradiation damage at high temperatures. It has been known for decades that high dose radiation can introduce significant void swelling accompanied by precipitation in austenitic stainless steel (SS). Here we report, however, that through nanoengineering, ultra-fine grained (UFG) 304L SS with an average grain size of ~100 nm, can withstand Fe ion irradiation at 500°C to 80 displacements-per-atom (dpa) with moderate grain coarsening. Compared to coarse grained (CG) counterparts, swelling resistance of UFG SS is improved by nearly an order of magnitude and swelling rate is reduced by a factor of 5. M_23_C_6_ precipitates, abundant in irradiated CG SS, are largely absent in UFG SS. This study provides a nanoengineering approach to design and discover radiation tolerant metallic materials for applications in extreme radiation environments.

Neutron irradiation responses of austenitic stainless steels (SSs) have been extensively studied for decades as these materials have broad applications as structural components in light water reactors and fuel cladding in fast spectrum nuclear reactors^1^,^2^,^3^,^4^. Under neutron irradiation, the microstructural damage in metals includes point defects, dislocation loops, voids, and precipitates, etc^5^,^6^,^7^,^8^,^9^,^10^. With high-dose (>10 dpa) neutron irradiation at elevated temperature (300–700°C), formations of voids and precipitates are the major microstructural changes in austenitic SSs^11^,^12^. Voids nucleated directly from the displacement spike, monovacancies or the supersaturation of vacancies^13^,^14^ can cause volumetric swelling (void swelling), which is widely observed in irradiated materials^15^. Significant void swelling in austenitic SSs has been reported decades ago^16^, and the swelling rate of austenitic SSs is much greater than its ferritic/martensitic counterparts^17^. Although advanced austenitic SS (such as Ti modified D9) has been developed, its void swelling resistance remains limited^18^, especially against the cladding performance demands of a fast spectrum reactor. Dramatic void swelling accompanied by the formation of precipitates in austenitic SSs jeopardizes their application as fuel cladding in advanced reactors^19^,^20^.

The design of advanced nuclear reactors calls for structural steels that can withstand hundreds of dpa under neutron radiation^18^. However typical water-cooled test reactors have a low accumulative neutron dose, 1–10 dpa/year. Thus heavy ion radiation has been increasingly used to produce microstructural damage in metals and ceramics for rapid simulation of fast neutron irradiation^21^,^22^,^23^, although high dose rate under heavy ion irradiation requires temperature shifts, compared to neutron irradiation, to produce comparable damage^24^. Formation of voids has been reported in heavy ion irradiated monolithic metals (for example, Ni^25^, Zr^26^ and Ge^27^), austenitic SSs (such as 316 SS^28^ and 304 SS^29^) and ferritic/martensitic steels^30^. Phase stability of austenitic SSs under neutron irradiation has been studied comprehensively. In 300 series austenitic SSs, irradiation-induced phases include γ′(Ni_3_Si), G phase (M_6_Ni_16_Si_7_) and phosphides, and irradiation enhanced/modified precipitates include M_6_C and M_23_C carbides^11^.

Internal defect trapping sinks redistribute the concentration of irradiation-induced point defects and their clusters, and thus have a significant impact on the formation of voids and phase stability under irradiation^13^,^31^. High angle grain boundaries (HAGBs), twin boundaries, phase boundaries and free surfaces can effectively absorb the radiation-induced defect clusters^32^,^33^,^34^,^35^,^36^,^37^,^38^,^39^,^40^. However, for nuclear reactor applications, these defect sinks need to be thermally stable against high temperature irradiations, ~500°C, mimicking the target fuel clad application temperature of certain fast reactors.

Severe plastic deformation (SPD) technique has been widely used to refine the microstructure of a variety of bulk metallic materials^41^,^42^,^43^. Here we used equal channel angular pressing (ECAP) technique to dramatically reduce the average grain size of austenitic 304L SS to ~100 nm. Our study shows that the ultrafine grained (UFG) 304 SS has excellent thermal stability (up to 600°C), and high strength as tested at 500°C. The stable defect sinks (high angle GBs) lead to substantial reduction of void swelling and swelling rate, comparable to some of the bench-marked ferritic/martensitic steels. This study thus provides an important step forward towards the design of advanced radiation tolerant structural steels with the assistance of nanoengineered stable defect sinks.

## Results

### Microstructure, thermal stability and tensile properties of UFG 304L SS

**Figure 1:**
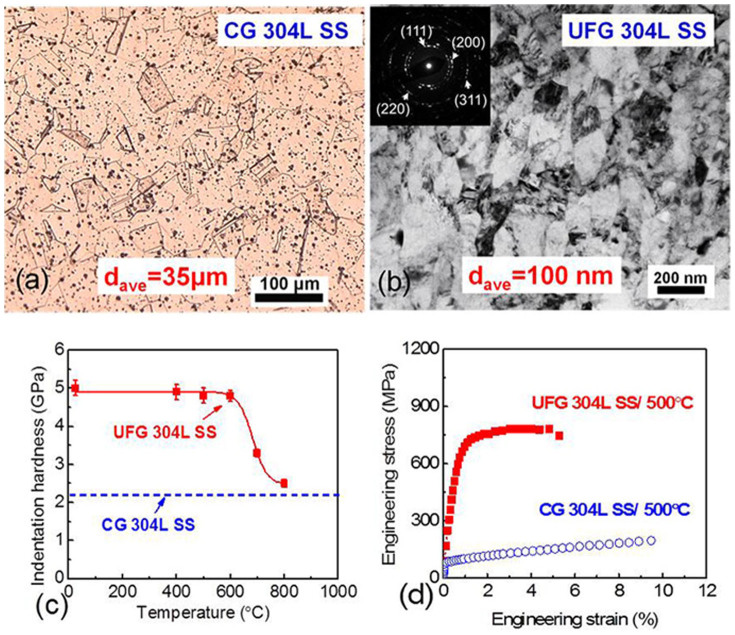
Microstructure, thermal stability and high temperature mechanical properties of ultrafine grained (UFG) 304L stainless steel (SS). (a) Optical micrograph of coarse grained (CG) 304L stainless steel showing an average grain size of 35 μm. (b) TEM micrograph of UFG 304 SS shows the average grain size is ~100 nm, and the inserted selected area diffraction (SAD) pattern shows austenite is the dominant phase. (c) Thermal stability of as-processed UFG 304L SS without radiation. The indentation hardness of the annealed specimens (one hour) measured at room temperature remained unchanged up to 600°C, followed by softening thereafter. (d) Engineering stress-strain curves of CG and UFG 304L SS under tension tests at 500°C. The 0.2% off-set yield strength is 630 MPa for UFG 304L SS, and 85 MPa for CG specimen.

The average grain size of coarse-grained (CG) SS as shown in an optical micrograph in Fig. 1a was ~35 μm. ECAP processing led to a dramatic reduction of the average grain size to ~100 nm, evaluated as the average value of the width and length of 150–200 grains, as shown in the transmission electron microscopy (TEM) micrograph in Fig. 1b. Fig. S1 shows the statistics of grain size distributions in CG and UFG SSs. The inserted selected area diffraction (SAD) pattern in Fig. 1b suggested the retention of fcc austenite as the primary phase after ECAP. Deformation-induced martensitic phase transformation in 304 SS typically occurs at ambient temperature. The temperature of ECAP experiments in this study was kept at ~500°C and no prominent martensitic phase transformation was observed. Thermal stability of the UFG SS was probed by nanoindentation hardness measurement on annealed specimens. Indentation hardness of UFG SS remained constant up to 600°C/1 h (vacuum annealing) as shown in Fig. 1c. Tensile tests were performed for CG and UFG SS at 500°C. The yield strength of UFG SS was 630 MPa, significantly greater than 85 MPa for as-received CG SS (Fig. 1d).

### Drastically enhanced void swelling resistance of UFG 304L SS

**Figure 2:**
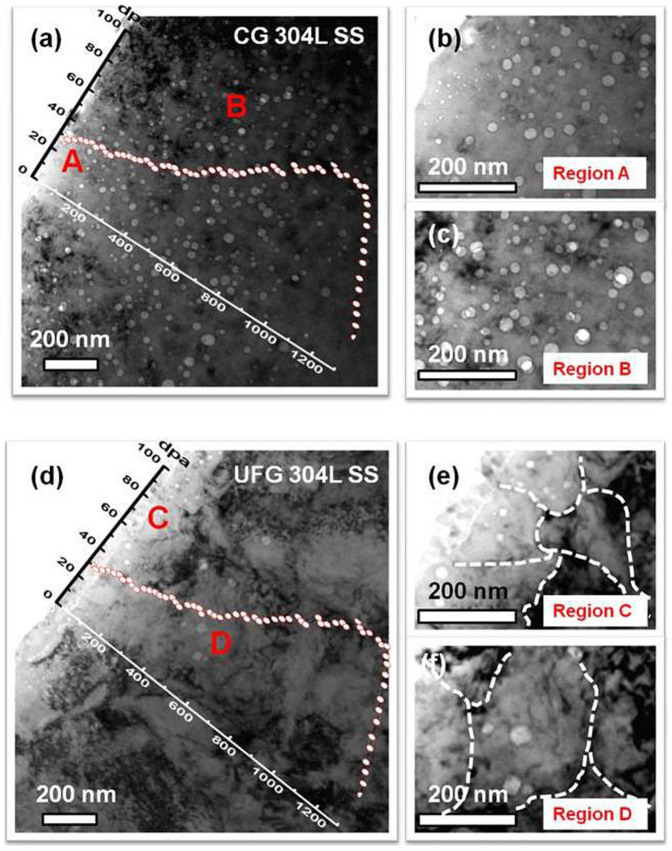
Extraordinary void swelling resistance of UFG 304L SS subjected to Fe ion irradiation at ion energy of 3.5 MeV and a total fluence of 6 × 10^20^/m^2^ at 500°C by defocusing the ion beam. (a) Panoramic cross-section TEM micrograph of Fe ion irradiated CG 304L SS showing a large number of voids. (b) The magnified TEM image of region A in Fig. 2a shows high-density small voids near the surface of irradiated CG 304L SS. (c) In region B of the same specimen, at a depth of ~500 nm from surface, high-density large voids were observed. (d) Cross-section TEM overview of irradiated UFG 304L SS showing much less voids. (e) The magnified TEM image of surface region e in irradiated UFG 304L SS shows numerous faceted voids distributed primarily along grain boundaries. (f) Magnified TEM micrograph of region f at ~500 nm from surface shows much lower void density compared to that in irradiated CG counterpart.

After 3.5 MeV Fe ion irradiation at 500°C up to a fluence of 6 × 10^20^/m^2^ (see Fig. S2 for SRIM simulation of depth dependent radiation dose), CG 304L SS had a large number of voids as shown in cross-sectional TEM micrograph in Fig. 2a. These voids have nearly identical characteristics compared to neutron radiation-induced voids as examined by using various focus condition (Fig. S3). Magnified TEM micrographs taken from surface region A (Fig. 2b) and region B (at a depth of 400–600 nm from surface in Fig. 2c) show a high density of voids. Conversely, in the irradiated UFG 304L SS, the panoramic TEM micrograph in Fig. 2d shows a sporadic distribution of voids. A magnified view of the irradiated surface region C (Fig. 2e) displays voids formed near free surface. In region D (Fig. 2f) the void density in UFG 304L SS is much lower than that in CG SS.

**Figure 3:**
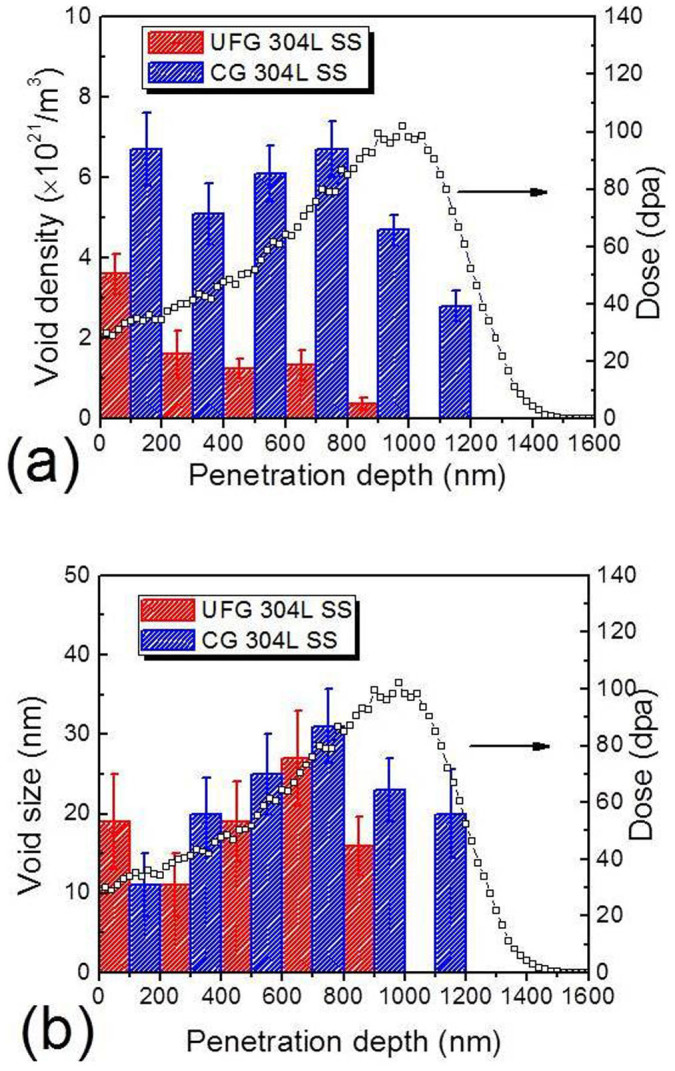
Statistic studies show that UFG 304L SS has significantly lower void swelling than CG 304L SS due to reduced void density and size. (a) Statistic analysis shows that void density along the projected radiation depth reached a maximum both near surface and at 700–800 nm in irradiated CG specimen. In UFG specimens, the maximum void density occurred near surface region, and the overall void density in UFG SS was much less than that of CG counterpart throughout the entire irradiated specimens. SRIM simulated depth dependent radiation damage (in unit of DPA) was superimposed on the same plot. (b) Distribution of void size along ion penetration depth shows that void size in UFG SS is in general comparable or slightly smaller than that in CG SS.

**Figure 4:**
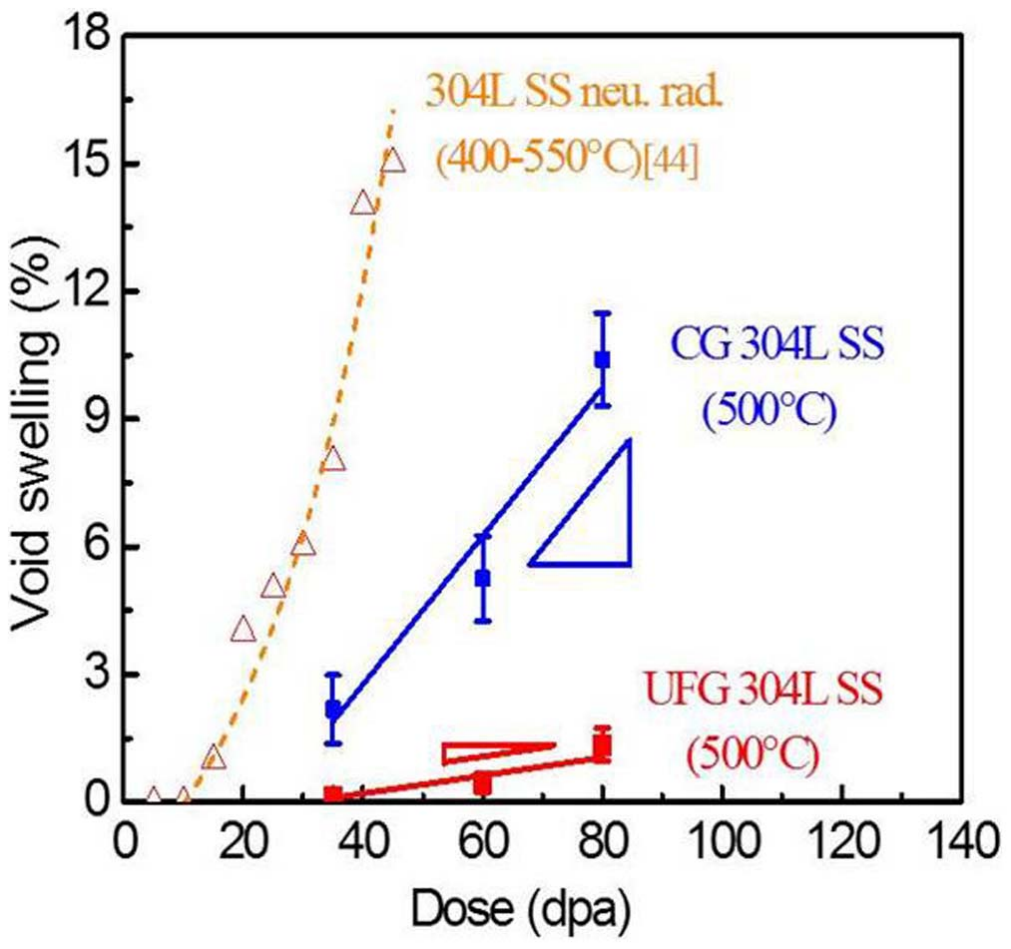
Comparison of void swelling of 304L SS irradiated by Fe ions (this study) and fast neutrons spectrum (in the literature [44]). UFG 304L SS has extraordinarily lower swelling and swelling rate than its CG counterparts.

Fig. 3a shows that void density along the projected radiation depth in irradiated CG SS reached a maximum both near surface and at 700–800 nm. In UFG specimens, the maximum void density occurred near the surface region, and the overall void density in UFG SS was much less than that of CG counterpart throughout the entire irradiated specimens. SRIM simulated depth dependent radiation damage (in unit of displacements-per-atom, dpa) was superimposed on the same plot. The average void size was similar in both irradiated CG and UFG SSs. The void density in the peak damage region of ECAPed sample is lower than other regions due to a prominent injection-of-interstitial effect in the peak cascade area. The injected interstitials recombine with vacancies and reduce the density of voids. The injected interstitial phenomenon has been reported in the literature^22^. To avoid deleterious surface and injection-of-interstitial effects on estimation of void welling, only the data (void density and size) obtained at a depth of 200–700 nm were taken into account for estimation of void swelling. As shown in Fig. S2, in the 200–700 nm region from surface, the damage level varies from 35 to 80 dpa, and the void density in CG SS is ~6.5 × 10^21^/m^3^, 5 times as much as that in UFG SS, ~1.3 × 10^21^/m^3^, consequently void swelling increased from ~2 to 10% in CG SS, comparing to ~0.1–1.5% in UFG SS (Fig. 4). A clear boundary was observed separating irradiated from non-irradiated regions in UFG 304L SS (Fig. S4). The average grain size of irradiated region is ~200 nm, compared to ~100 nm in non-irradiated area.

Fig. 4 compares the void swelling of Fe ion irradiated 304L SS (this study) with neutron irradiated 304 in literature^44^. At a similar radiation dose, 80 dpa, void swelling is ~10% for CG 304L SS, while UFG 304L SS has a sharply lower void swelling, ~1.3%, nearly an order of magnitude lower than its CG counterpart. In addition, the average void swelling rate over 35–80 dpa was ~0.18%/dpa in CG SS vs. ~0.03%/dpa in UFG SS. Weak beam dark field (WBDF) TEM studies show that although dislocation loop density in irradiated CG 304SS is slightly greater than those in UFG SS, the CG SS has an average loop size of ~25 nm, much greater than that in UFG SS, ~8 nm (See supplementary Fig. S5).

### Suppressed formation of precipitates in UFG 304L SS

**Figure 5:**
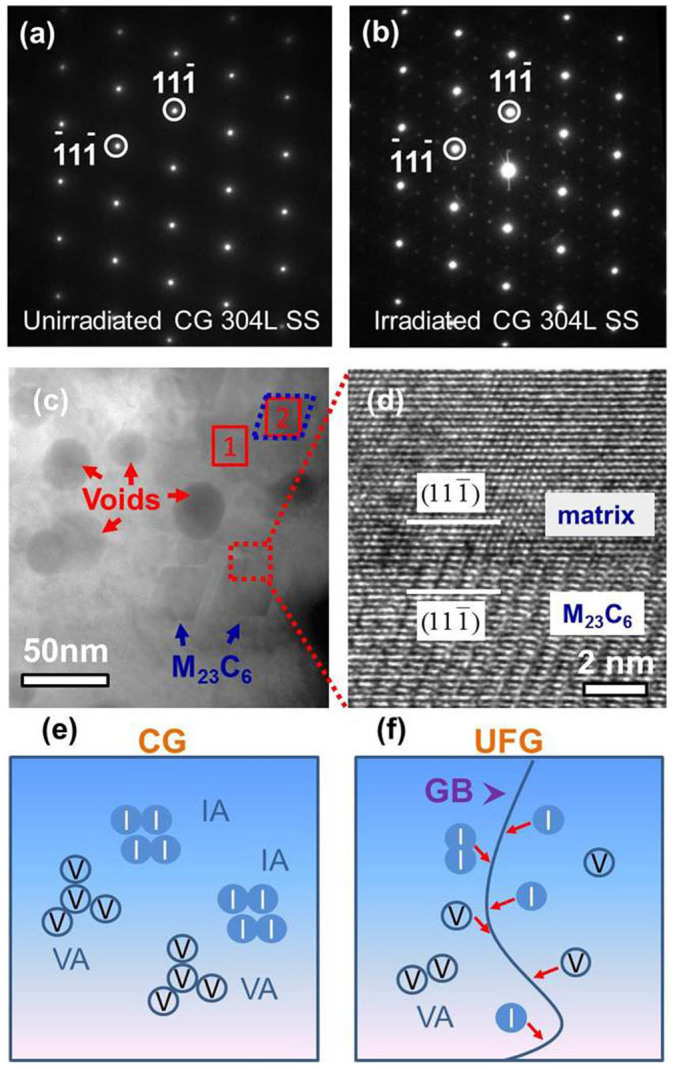
Fe ion irradiation-enhanced precipitation in CG 304L SS. (a) SAD pattern of unirradiated CG 304L SS shows classical single fcc phase examined along [011] zone axis. (b) SAD pattern of Fe ion irradiated CG 304L SS (along the identical zone axis) displays superlattice diffractions arising from M_23_C_6_ precipitates. The d-spacing of precipitates was about three times as large as that of the matrix. (c) Scanning transmission electron microscopy (STEM) image of the irradiated CG SS shows numerous precipitates (M_23_C_6_) adjacent to spherical voids. (d) HRTEM micrograph of the {111} phase boundary between matrix and a M_23_C_6_ precipitate. (e) Schematic illustration of formation of precipitates in CG sample under irradiation. Interstitial aggregates (IAs) and vacancy aggregates (VAs) are created. IAs can form interstitial loops or precipitates and VAs can form vacancy loops or voids. (f) In irradiated UFG sample, interstitials and vacancies migrate towards the grain boundaries at elevated temperature and thus suppress the formation of IAs, precipitates and voids.

Comparing to the SAD pattern of unirradiated CG SS (Fig. 5a), superlattice diffraction from irradiated CG SS (Fig. 5b) originates from precipitates. The lattice parameter of precipitate was 1.042 nm, about three times as large as that of the matrix, 0.351 nm. Scanning transmission electron microscopy (STEM) micrograph of irradiated CG SS in Fig. 5c shows the coexistence of voids and precipitates. HRTEM image (Fig. 5d) reveals the typical {111} phase boundary between matrix and precipitates. Chemical analyses (Fig. S6) suggest that precipitates in irradiated CG SS are M_23_C_6_.

## Discussion

### Mechanisms for superior swelling resistance of UFG SS

There are numerous challenges facing the application of austenitic SSs for advanced nuclear reactors, among which void swelling and precipitation are two major concerns. Austenitic SSs have notoriously poor resistance to void swelling (greater than 100% has been observed previously^45^). For practical applications in advanced nuclear reactors, however, curtailing swelling to less than a few volume % is critical to maintain mechanical and structural stability of fuel cladding materials. Ferritic/martensitic steels have been shown to possess superior swelling resistance compared to their austenitic counterparts. Here we show by grain refinement to 100 nm, UFG 304 SS has accomplished outstanding swelling resistance (<2%) up to 80 dpa at 500°C. The superior void swelling resistance in UFG SS arises from the following mechanisms.

**First**, defect removal due to the introduction of ample GB defect sinks. Sink strength of GBs (*S_gb_*) in UFG 304L SS is estimated to be ~6 × 10^15^/m^2^, drastically greater than that in CG SS, ~5 × 10^12^/m^2^ (see supplementary information for detailed calculations). GBs are generally considered as neutral sinks for radiation induced point defects. At elevated temperature (500°C), GBs can effectively absorb both interstitials and vacancies, and thus increase the possibility of their recombination along the GBs as illustrated in Fig. 5 e–f. It follows that GBs lower the concentration of vacancy clusters, and thus mitigate the formation of voids. Through *in situ* Kr ion irradiation studies, Sun *et al*^32^ showed that GBs in nanocrystalline (nc) Ni can effectively absorb dislocation loops and dislocation segments. The vacancy concentration near GB is lower than that in grain interior. Such a vacancy concentration gradient accelerates the migration of dislocation loops towards GB and enhance defect capture rate. Han et al.^46^ reported that void denuded zone is mostly related to the grain boundary characteristics. In ion irradiated CG Cu, for the non-Σ3 GBs, the width of the void denuded zone generally increases with misorientation angle. In the current UFG SS, the number density of voids in the irradiated region appropriate for statistic studies (at a depth of 200–700 nm) is too low to procure reliable statistics on the GB character dependent distribution of voids.

**Second**, high density of dislocations generated during severe plastic deformation (~5 × 10^15^/m^2^) could also alleviate the formation of defect clusters. The sink strength of dislocations *S_disl_* (proportional to dislocation density) is estimated to be ~5 × 10^15^/m^2^ for UFG and ~1 × 10^14^/m^2^ for CG SS. Although dislocations are typically considered as biased defect sinks (preferentially absorbing interstitials), such dislocation networks adjacent to GBs could facilitate rapid transportation of point defects towards GBs, where opposite types of point defects annihilate.

**Third**, The kinetic rate theory^47^ predicts that cavity growth rate in irradiated steels is related to the sink strength ratio of dislocation (*S_disl_*) and GB (*S_gb_*) to voids, Q, which can be expressed as 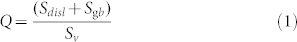
where *S_v_* is the sink strength of voids, which scales with the density and size of voids. In general when either Q ≫ 1 (that is GBs and dislocations are dominant defect sinks), or Q ≪ 1 (that is voids prevail), the void swelling rate is much lower than that when Q ≈ 1–10 (where sinks are more balanced)^47^. Although the number density of voids in CG 304L SS is ~6 × 10^21^/m^3^, greater than that of UFG SS, ~1 × 10^21^/m^3^, the Q ratio is ~67 for UFG 304L SS, ≫1, comparing with ~0.5 for CG 304L SS (see supplementary table S1). Hence this analysis based on kinetic rate theory agrees qualitatively with our experimental observations of excellent void swelling resistance in UFG SS.

**Fourth**, thermal stability of UFG grains under radiation at elevated temperature. Grain coarsening is a major threat for the application of nc metallic materials at elevated temperature. The retention of high density GBs ensures sustainable and reliable sources of defect sinks for the removal of radiation induced defect clusters. This is extremely important as defect sinks, such as dislocations, are typically unstable (manifested as a reduction of dislocation density) after high dose radiation at such a temperature (500°C). The current study shows that UFG grains exhibit only moderate grain growth after an extended period of high dose radiation at 500°C.

The thermal stability of UFG 304L SS could arise from the following mechanisms. First, the UFG 304L SS has numerous minute solutes, some of which could segregate to HAGBs, and provide GB drag force that retards grain growth. Second, the selected area diffraction shows incomplete diffuse rings, indicating that not all GBs are of high angle characteristics. Low angle GBs or subgrain boundaries may have greater thermal stability than their HAGB counterparts. Third, the average grain size of UFG SS is ~100 nm, much greater than some of the nanograins (~tens of nm or less) in monolithic metals, thus making UFG SS less vulnerable to grain coarsening at elevated temperatures. To apply UFG 304L SS at even higher temperature for advanced fast reactors, the thermal stability of UFG 304L SS needs to be enhanced even further. Approaches such as introduction of stable second phase particles, and more effective solutes to GBs could be implemented for such purposes.

Comparison of dose dependent void swelling among several austenitic SSs shows that heavy ion irradiation may not exactly replicate the microstructures generated by neutron radiation. The Fe ion irradiation at 500°C in current study is comparable to fast neutron irradiation at 390°C, calculated by the temperature shift of swelling with dose rate^47^. Numerous factors make such direct comparisons more complicated. First, in Fig. 4, the void swelling of neutron irradiated CG304L SS is more significant than that in Fe ion irradiated CG 304L SS. Transmutation of Helium during neutron irradiation can accelerate the void swelling, while there is no transmutation reaction during ion irradiation. Second, the dose rate of neutron irradiation is much lower than that of heavy ion irradiation introduced in this study. Nevertheless, the general trend of UFG enabled suppression of void swelling sustains as both sets of specimens were irradiated at the same conditions. Furthermore the magnitude of void swelling in heavy ion irradiated CG specimen is not too different from that of neutron irradiated counterparts.

### Mitigation of precipitation in irradiated SS by grain refinement

In 300-series austenitic SSs, several types of precipitates can form under irradiation. G phases arising from radiation-induced segregation (RIS) were observed on GBs in a titanium-modified austenitic SS under fast neutron irradiation at 420°C^48^. Radiation-enhanced precipitates in the form of M_23_C_6_ carbides were found in neutron irradiated 316 SS (up to 44 dpa) and FV548 steel at a dose of 30 dpa^49^. The formation of the brittle carbides reduces fracture toughness of irradiated materials. The segregation of Cr to the carbide also reduces the corrosion resistance of irradiated steels. In this study, in irradiated CG 304L SS, M_23_C_6_ carbides were frequently observed adjacent to large spherical voids, implying the preferential segregation of solute atoms may be directly tied to the formation of voids (e.g. acceleration of their formation). In the literature, no intragranular extended precipitates were observed in Fe ion irradiated nanostructured 316 SS up to 10 dpa at 350°C^50^. What is striking in this study is that in Fe ion irradiated CG 304L SS, precipitates were formed, while in irradiated UFG 304L SS, precipitates were largely absent up to ~80 dpa at 500°C. The nucleation of M_23_C_6_ precipitates was suppressed by the abundant HAGBs in UFG 304L SS. This might be attributed in part to the migration of radiation-induced point defects and defect clusters towards GBs (due to lower point defect supersaturation at GBs), which would limit clustering of solutes in the grain interior as shown schematically in Fig. 5 e–f. The reduced sink strength of voids in the matrix would also contribute to less preferential segregation of solutes within grains for similar reasons. It is also important to realize that the formation of precipitates adjacent to voids in CG 304L SS could also accelerate swelling in irradiated materials as shown in prior studies^11^. In this sense, the effective suppression of precipitation clearly has positive impact in alleviating void swelling in irradiated UFG 304L SS.

In summary, we demonstrate a simple yet effective strategy, nanoengineering of grains, which can dramatically reduce the magnitude of void swelling of irradiated 304L SS to unprecedented level, by nearly an order of magnitude. UFG grains also effectively alleviate the formation of carbide precipitates. The UFG grains in 304L SS also have exceptional thermal stability against radiation at elevated temperature and enable a combination of high strength and good ductility. The present study implies that UFG austenitic SSs have promising applications in extreme radiation environments. The use of nanograins in combination with other defect sinks, such as high densities nanoscale oxide or nitride precipitates could lead to even greater improvement in radiation resistance. As radiation damage (such as swelling) is a universal phenomenon (also observed in Si)^15^, the current study may also have implications for the design of radiation resistant materials with self-healing capability for electronic device in extreme environments.

## Methods

The average grain size of as-received CG 304L SS, as shown in Fig. 1a, was ~35 μm. Equal channel angular pressing (ECAP) technique was performed (up to 4 passes at 500°C with Route Bc) to refine the microstructure of the SS. Thermal stability of UFG 304L SS was studied by probing indentation hardness evolution after vacuum annealing at various temperatures (up to 800°C) for one hour. Indentation hardness was measured at room temperature at an indentation depth of 1 μm by using a Fischerscope HM 2000XYp nano/microindentor with a Vickers diamond indenter, using instrumented nanoindentation technique similar to the continuous stiffness measurement method as described by Oliver and Pharr^51^. Uniaxial tensile tests at constant strain rate of 1 × 10^−3^/s were performed at 500°C for CG and UFG 304L SS.

Fe ion irradiations at energy of 3.5 MeV were performed at 500°C for CG and UFG 304L SS to a fluence of 6 × 10^20^ ions/m^2^ with a defocused beam without raster scanning. The heating mechanism used in this study is a light-bulb heater and read-off is Omega HH378 with K-type thermal-couples. The thermal-couples were mounted on the stage surface less than 5 mm away from the irradiation area, and avoided direct ion beam radiation. The overall temperature fluctuation during ion irradiation was controlled within 10°C. The average dose rate was ~0.003 dpa/s. The stopping and range of ions in matter (SRIM) method^52^ was used to predict the damage profile along the penetration depth by Kinchin-Pease method, as shown in Fig. S2, and the displacement energy 25 eV was used in the calculation. High resolution transmission electron microscopy (HRTEM) and scanning transmission electron microscopy (STEM) with energy dispersive spectroscopy (EDS) experiments were performed on an aberration corrected Titan transmission electron microscope operated at 300 kV and equipped with a Gatan SC1000 ORIUS CCD camera. Cross-section TEM (XTEM) samples were prepared by focused ion beam (FIB) technique, which did not introduce phase transformations in specimens. Artifacts induced by FIB were removed by ion polishing with low current (60 pA) and low voltage (5 kV) during the final stage of thinning.

## Author Contributions

C.S. and K.T.H. processed the UFG 304L SS. C.S. and S.Z. performed the electron microscopy studies. C.C.W. and L.S. performed Fe ion irradiation experiments. Y.W. and Y.Y. contributed to FIB-TEM studies. S.A.M., S.J.Z., T.R.A. and H.W. assisted with interpreting the data. X.Z. developed the concept and directed the project. All authors discussed the results and commented on the manuscript.

## Supplementary Material

Supplementary Information Supplementary Information
