# Supplementary material for: Superior radiation-resistant nanoengineered austenitic 304L stainless steel for applications in extreme radiation environments
Source: Sci Rep. 2015 Jan 15;5:7801. doi: 10.1038/srep07801 (PMC4295098; doi:10.1038/srep07801)
Supplement: Supplementary Information [file srep07801-s1.doc]

**Supplementary Information**

**Superior radiation-resistant nanoengineered austenitic 304L stainless steel for applications in extreme environments**

C. Sun^1,2^, S. Zheng^2^, C. C. Wei^3^, Y. Wu^4^, L. Shao^3^, Y. Yang^4^, K.T. Hartwig^1^,

S. A. Maloy^2^, S. J. Zinkle^5^, T. R. Allen^6^, H.Wang^7^, and X. Zhang^1,8*^

^1^Department of Materials Science and Engineering, Texas A&M University, College Station, TX 77843

^2^ Materials Science and Technology Division, Los Alamos National Laboratory, Los Alamos, NM 87545

^3^Department of Nuclear Engineering, Texas A&M University, College Station, TX 77843

^4^Department of Materials Science and Engineering, Nuclear Engineering Program, University of Florida, Gainesville, FL 32611

^5^Metals and Ceramics Division, Oak Ridge National Laboratory, Oak Ridge, TN, 37831, USA

^6^Department of Engineering Physics, University of Wisconsin, Madison, WI 53706, USA

^7^ Department of Electrical and Computer Engineering, Texas A&M University, College Station, TX 77843

^8^Department of Mechanical Engineering, Texas A&M University, College Station, TX 77843

*Corresponding author: X. Zhang, [zhangx@tamu.edu](mailto:zhangx@tamu.edu), Tel: (979) 845-2143

- **Supplementary figures**

Fig. S1. Grain size distribution for unirradiated CG and UFG 304L stainless steel (SS). (a) The average grain size of CG 304L SS is 35µm; (b) The average grain size of UFG 304L SS is ~100 nm.

Fig. S2. Depth profile of radiation damage (dpa) in Fe-18Cr-8Ni alloy (304 SS) subjected to Fe ion irradiation at 3.5MeV with a fluence of 6×10^20^/m^2^ obtained from SRIM simulation by using Kinchin-Pease method. For void swelling analysis, to avoid the surface effect and influence of injected interstitial in the peak damage region, radiation damage between 200-700 nm was used for void swelling analysis.


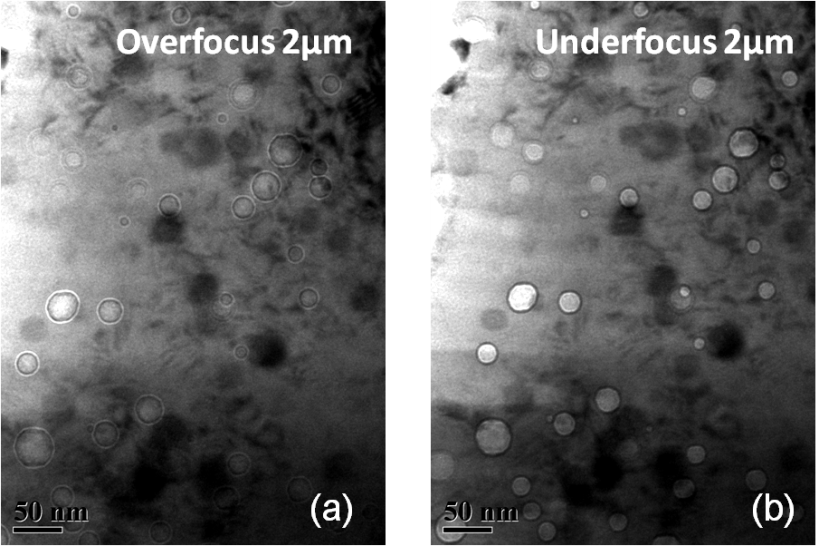


Fig. S3. Examination of voids in irradiated CG 304L SS by using different focus conditions. (a) Overfocus +2 μm. (b) Underfocus -2 μm. Fresnel fringes surrounding voids swap their contrast when varying from over- to under-focus conditions.


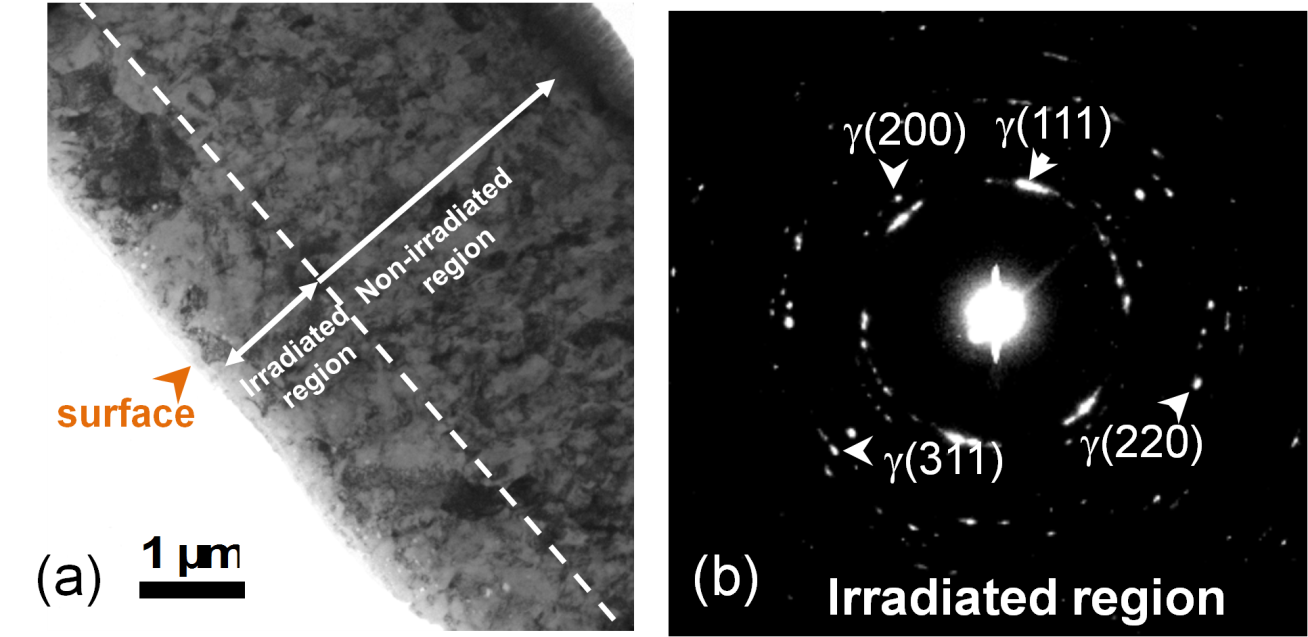


Fig. S4. (a) Low magnification TEM image of UFG 304L SS under irradiation at 500°C. Grain growth was observed in the irradiated region. A clear boundary between irradiated and non-irradiated region is visible and marked by a dash line. (b) SAD pattern of the irradiated region in UFG sample, showing no reflections from the precipitates. (c) Statistics of grain size in non-irradiated region shows the average grain size is ~ 100 nm. (d) Under irradiation, the average grain size increased to ~200 nm.


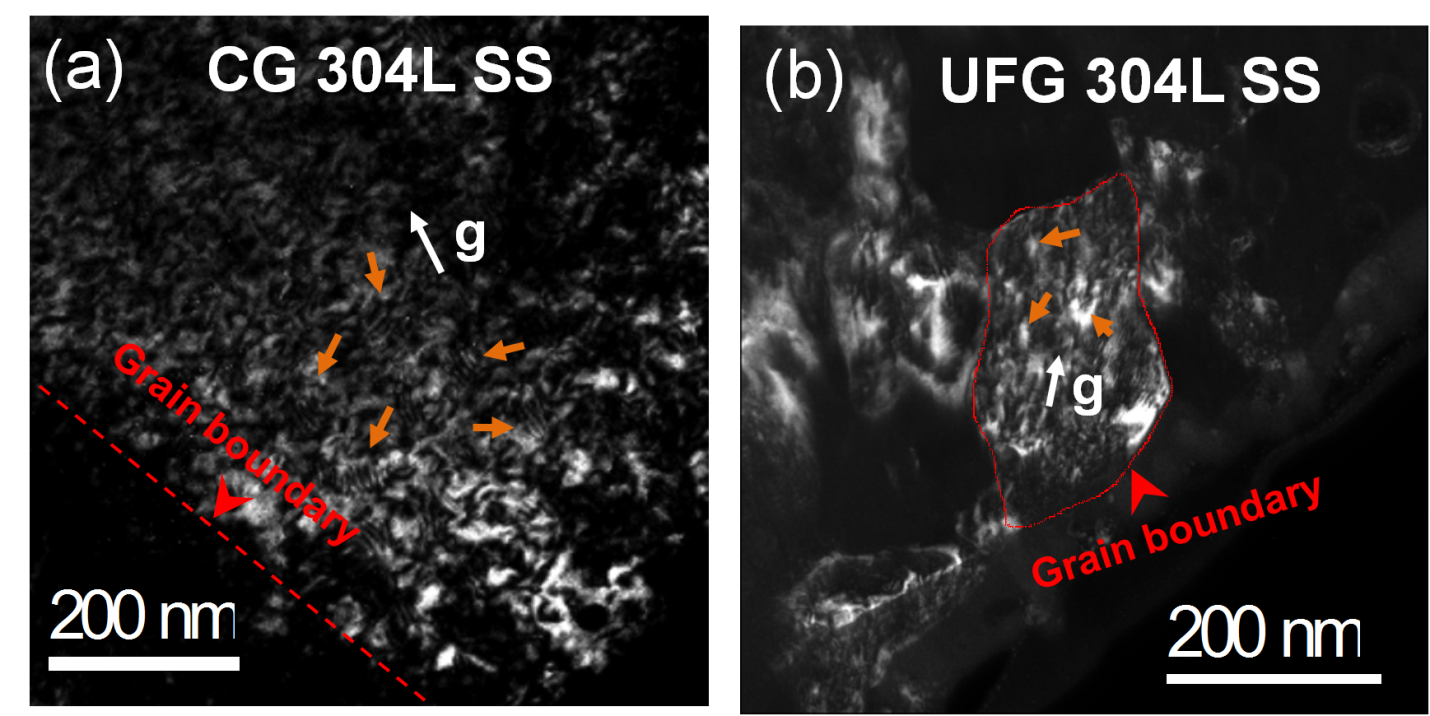


Fig. S5. Dark field TEM image of 304L SS under Fe ion irradiation at 500°C with diffraction vector g = [111]. The inserted orange arrows indicate the dislocation loops. (a) In irradiated CG 304L SS, the average size of dislocation loop is ~ 25 nm and the number density is ~4×10^21^/m^3^. The density of dislocation segment is ~ 1×10^14^/m^2^. (b) For irradiated UFG 304L SS, the average size of dislocation loops is much smaller, ~ 8 nm and their number density is ~ 3×10^21^/m^3^. No clear dislocation segments were observed in irradiated UFG 304L SS.


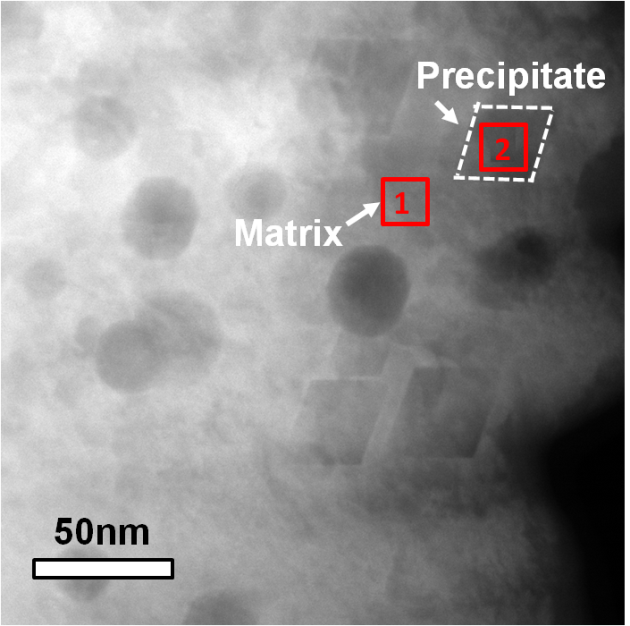


| **Weight percentage (%)** | **Fe** | **Ni** | **Cr** | **C** |
| --- | --- | --- | --- | --- |
| Area 1 | 79.4 | 5.6 | 14.2 | 0.8 |
| Area 2 | 49.7 | 9.3 | 37.9 | 3.1 |

Fig. S6. Chemical analysis of irradiation-induced precipitate in CG 304L SS by STEM/EDS. (a) STEM image of the irradiated CG specimen. The white dash lines outline the boundary of a precipitate. (b) EDS spectra of the matrix (box 1) and precipitate (box 2) show the concentration of various elements, and quantitative compositions (in weight percentage) are shown in the succeeding table. The precipitate is enriched in Cr and C, implying its carbide nature.

- **Calculations of sink strength to predict void swelling resistance of irradiated 304L SS**

The kinetic rate theory predict that cavity growth rate in irradiated steels is related to the ratio of dislocation to void sink strength, Q, which can be expressed as

$\text{Q=}\frac{\text{S}_{\text{disl}}}{\text{S}_{\text{v}}}$ (1).

The sink strength of void (S_v_) and sink strength of dislocations (S_disl_) can be calculated as follows:

1. **Sink strength of voids ($S_{v}$)** :

(2),

where $r_{v}$ and $N_{v}$are radius and number density of voids, respectively. At depth of 600nm, $r_{v}=13\text{nm}$ and in CG sample and in UFG sample. $Z_{c}$is the cavity sink capture efficiency of order unity.

1. **Sink strength of dislocation ($S_{\text{disl}}$)** :

(3),

where $Z_{d}$is the dislocation capture efficiency of order unity and $\rho_{\text{disl}}$is the density of dislocation, including preexisting dislocations, dislocation loops and segments.

Notice that grain boundaries are similar defect sinks comparing to dislocations, thus

Equation (1) is modified as follows:

$\text{Q=}\frac{\text{(S}_{\text{disl}}\text{+S}_{\text{gb}}\text{)}}{\text{S}_{\text{v}}}$ (4),

The kinetic rate theory predicts that when Q >> 1 (that is GBs and dislocations are dominating defect sinks), or when Q << 1 (that is voids dominate), the void swelling rate is much lower than the case when Q ≈ 1-10 .

The sink strength of GBs is estimated as follows:

1. **Sink strength of grain boundaries ($S_{\text{gb}}$)** :

$S_{\text{gb}}=60/{d^{2}}$, when (5),

, when (6),

where d is the grain diameter, $d$=100 nm and 35 μm for UFG and CG sample. $S$is the cumulative sink strength of all sinks and assumed to be 1×10^15^/m^2^.

The following table summarizes the calculated sink strength for various type of defects in UFG and CG 304L SS, as well as Q values. It is evident that for UFG SS, Q is ~ 67, >> 1. Thus GBs are dominating defect sinks, which enable extraordinary void swelling resistance in UFG SS. In contrast in CG 304L SS, Q is ~ 0.5, implying that defect sinks (voids versus dislocations) are more balanced and hence the sink strength is very limited, rendering poor void swelling resistance.

**Table S1. Summary of sink strength of various types of sinks in current study**

|  | Grain boundary $S_{\text{gb}}$ ( m^-2^) | Dislocation,$S_{\text{disl}}$ ( m^-2^) | | Void $S_{v}$ ( m^-2^) | $\text{Q=}\frac{\text{(S}_{\text{disl}}\text{+S}_{\text{gb}}\text{)}}{\text{S}_{\text{v}}}$ |
| --- | --- | --- | --- | --- | --- |
|  |  | Preexisting dislocations $S_{1}$ ( m^-2^) | Radiation-induced dislocations $S_{2}$ ( m^-2^) |  |  |
| UFG 304L  Stainless Steel | 6×10^15^ | 5×10^15^ | 0.8×10^14^ | 1.5×10^14^ | ~ 67 |
| CG 304L  Stainless Steel | 0.5×10^13^ | 1×10^14^ | 4×10^14^ | 1×10^15^ | ~ 0.5 |

**Reference**

1. L. Mansur, Theory and experimental background on dimensional changes in irradiated alloys. *J. Nucl. Mater.* **216**, 97 (1994).

2. S. J. Zinkle, L. L. Snead, Designing Radiation Resistance in Materials for Fusion Energy. *Annual Review of Materials Research* **44**, null (2014).
